# Defining child health in the 21st century

**DOI:** 10.1038/s41390-024-03423-w

**Published:** 2024-08-26

**Authors:** Ruth E. K. Stein

**Affiliations:** Professor of Pediatrics Albert Einstein College of Medicine and Children’s Hospital at Montefiore, 1300 Morris Park Avenue, VE 6B27, Bronx, NY 10461 USA

## Abstract

**Abstract:**

The concept of child health has evolved over many decades and has gone from defining health as the absence of disease and disability to a much more sophisticated understanding of the ways in which a confluence of many factors leads to a healthy childhood and to producing the infrastructure for a healthy lifetime. We review the evolution of these ideas and endorse the definition featured in *Children’s Health, the Nation’s Wealth*, which states tha*t* child health is: “… the extent to which individual children or groups of children are able or enabled to: (a) develop and realize their potential, (b) satisfy their needs, and (c) develop the capacities that allow them to interact successfully with their biological, physical, and social environments.”

**Impact:**

- The definition of child health and the model presented form a framework for conducting and interpreting research in child health and understanding the ways in which influences affect child health.
- They also demonstrate how child health is the foundation for life-long health.
- Child health is dynamic and is always changing.
- There are many influences affecting child health at any given time.
- Because each child’s health is different, they may react in distinctive ways to a new health challenge.

This model is best represented by a kaleidoscope of influences (biology, social and built environment, behavior, policies, and services) working together over a child’s life and developmental trajectory. The model has life-long implications for adult health and well-being and has far-reaching implications for promoting children’s health and for understanding research in child health. Pediatrics is a field devoted to improving the health of children, but what does that really mean? There are several aspects to this all-important question. How do we currently view and define child health? How do we understand the things that underpin a healthy childhood? What is the significance of child health for life-long health? The answers to these questions are important for all our endeavors as child-oriented clinicians and are key to our ongoing research efforts to improve child health.

## Approaches to child health

The concept of child health has evolved over many decades and has gone from defining health as the absence of disease and disability to a much more sophisticated understanding of the ways in which a confluence of many factors leads to a healthy childhood and to producing the infrastructure for a healthy lifetime. While this is true in the United States and most of the upper income nations and the elite in many other communities, many low- and middle-income countries still experience high childhood morbidity and mortality and low rates of immunizations that protect children from many diseases. Thus, for many of them, the absence of disease continues to be a major marker of improvements in child health.

The recognition that health is more than the absence of disease was relatively novel when it was written into the constitution of the World Health Organization (WHO) in 1945. In April 7, 1948, the WHO Charter was adopted and definition was formally recognized. It stated “Health is a state of complete physical, mental, and social well-being and not merely the absence of disease or infirmity” (https://www.who.int/about/accountability/governance/constitution). There were several other rather revolutionary components to the constitution, including the notion that health was a human right, a call for equity in health, and the statement that governments had a responsibility for insuring health (https://www.who.int/about/accountability/governance/constitution). Unfortunately, the latter two elements are still not always accepted as fundamental in the United States.

An often-overlooked component of the WHO Charter is the principle that: “Healthy development of the child is of basic importance; the ability to live harmoniously in a changing total environment is essential to such development” (https://www.who.int/about/accountability/governance/constitution). This last principle is the only component that mentions development, something that is fundamental to childhood and yet was not incorporated into most people’s thinking about health at the time. It should be emphasized that there was little thought to differentiating the definitions of health for children and adults.

Over the next several decades, the focus was on the elimination of diseases, something that became possible with the explosion of new biomedical understanding of the causes and mechanisms for disease and the morbidity and mortality that they caused. This was a period during which there was development of a library of antibiotics and vaccines to treat and prevent many infectious diseases. The dominance of biomedical sciences resulted in a focus primarily on aspects of physical health, with emotional, cognitive, or social well-being being relatively ignored during that period. As a result, the notion that health was more than the absence of diseases got relatively little attention and a biomedical model of health dominated most discussions, whether focused on children or adults.

The mid 1970’s saw an explosion of new thoughts about child health and the key role of child development. Major elements of this new thinking were the increasing focus on differences in the biology of children and adults and in the appreciation of the extent to which factors outside of biology influenced health, especially among young children. In terms of appreciating differences between child and adult health, there was increasing recognition of the rapidly changing nature of children’s physical and behavioral characteristics; of their inherent dependency, especially early in life, and the differences in their exposure to environmental hazards and in the way that manifest poor health. The growing awareness of their biological differences led to recognition of such variations as their relative surface area, their play which placed them in closer proximity to ground level pollutants, and a myriad of metabolic differences that pediatric research had revealed.

Additionally, there was growing data on a variety of key factors other than the child’s vulnerability to infectious disease that were coming to the fore. One was the importance of infant attachment for healthy development; another was the recognition of how environmental factors such as lead were detracting from healthy development and well-being, and a third was recognition of child abuse and neglect by Henry Kempe.^1^ In addition, the implementation of Medicaid and the Children and Youth projects had brought many underserved communities into traditional medical venues, where clinicians were increasingly recognizing the impact of poverty on child health. These ideas were crystalized in Robert Haggerty’s studies of *Child Health and the Community*, published in 1975,^2^ which examined health care in Monroe country, a microcosm of the US population and focused on many factors in the lives of children beyond their biology and exposure to infectious diseases.

In response to these trends and to the increasing recognition of non-infectious causes of disease, three new broad models were put forward, each of which made major contributions to the conceptualization of child health.

The first of these was from George Engel, a psychiatrist at the University of Rochester, who proposed a revolutionary concept, which he called the “biopsychosocial” model of child health.^3^ His thesis was that biological, psychological, and social components each contributed to health and that all these factors had to be considered.^3^ The notion that the body and mind were connected was not entirely new and has been cited as going back at least to Descartes, but had been neglected during the focus on biology as the primary cause of illness. Engel reintroduced the concept that the social and psychological contributions to illness and well-being needed to be considered in medical science and health care, both as causes of disease and as important in their treatment.^4^ Broadening the notion of health to include factors outside of biology required consideration of how these elements interacted. Engel’s model was a Venn diagram with overlap of the three types of factors: biological, social, and psychological factors. The interaction of these elements resulted in the manifestations of illness and the elements that needed to be considered in approaching care and understanding health.

At about the same time, a second set of ideas about how these factors affected one another came from a psychologist, Arnold Sameroff,^5^ who developed the notion of factors relating to health affected one another in a transactional way. He proposed that the parent, child, and environment interact in ways in which each affects the other and it is the sum of those interactions that leads to the child’s development and affects the child’s health and well-being. The notion of reciprocity and interactions of multiple factors remains key in thinking today.^5^

The third idea was the ecological model developed by Uri Bronfenbrenner, also a psychologist, who proposed a series of systems that influence one another and in total affect child health and development.^6^ The five components ranged from the microsystem (the child’s relationship with his or her immediate environment, school, and family) to macrosystems (culture, economy, customs, and bodies of knowledge). Each layer of the environment was visualized as a concentric circle, with the child in the middle. To a large degree it was his thinking about the broader set of factors that impact child health that has stayed with us and has helped us to think beyond the child’s immediate context when considering influences on health. Bronfenbrenner’s view of the way that environment interacted with the child’s health and development dominated for many years.^6^

After these three models were proposed, there was little innovative thinking about child health for a rather long period. During the ensuing decades most people accepted that the context in which a child was growing impacted his or her mental and physical health and contributed to well-being. An increasing number of studies focused on the broader issues affecting child health and on how these issues altered the manifestations of health and the outcomes of treatment. Yet none of this thinking led to a reformulation of how to define child health.

## Current definition

In 2001, at Congress’s request the Office of Disease Prevention and Health Promotion of the United States Department of Health and Human Services funded the Board of Children, Youth, and Families (BoCYF) of the National Research Council and the Institute of Medicine to do a study to assess the ways that child health was monitored in the United States and to make recommendations about ways to improve its measurement.^7^ This Committee on the Evaluation of Children’s Health: Measures of Risk, Protective and Promotional Factors for Assessing Child Health in the Community was charged with examining what was known about child health, the risk and protective factors and how the assessment of child health could be improved. The BoCYF convened a multidisciplinary committee to conduct the study. The first step that the committee undertook was to define child health and to do so it looked at available definitions of health. The committee noted that in general definitions of child health were not distinct from those for adult health. The WHO definition, as modified by the Ottawa Convention was the primary definition available. In the Ottawa Convention the term health was viewed as “the extent to which a group or individual can fulfil their ambitions and needs, on the one hand, and evolve with or adapt to the environment, on the other” (https://www.who.int/teams/health-promotion/enhanced-wellbeing/first-global-conference). It further stated that “Health is thus seen as a resource for everyday life, not as the goal of life; it is a positive concept that emphasises [sic] social and individual resources as well as physical capabilities. Thus, health promotion is not just a health issue, but goes beyond healthy lifestyles to well-being” (https://www.who.int/teams/health-promotion/enhanced-wellbeing/first-global-conference). This was the first time promotion of health was specifically advocated by a large number of countries.^8^

In examining the Ottawa Convention definition, the committee became aware that there were no clear references to the notion of development, which is such a critical component of child health and a fundamental concept in pediatrics. This is because using the WHO and Ottawa definitions, an individual who did not develop at all after birth might be considered entirely healthy–something most people would not agree with.

Based on the special characteristics of children’s health and the prior definitions, “the committee sought a comprehensive and integrative definition and conceptualization of health that reflects the dynamic nature of childhood, is conceptually sound, is based on the best scientific evidence, and provides a basis for both measuring and improving child health.”^7^ (page 32) Further, it recognized “that health and well-being are a result of interactions of many biological, psychological, social, cultural, and physical factors.”^7^ (pages 32 and 33)

The committee defined child health:

“… as the extent to which individual children or groups of children are able or enabled to: (a) develop and realize their potential, (b) satisfy their needs, and (c) develop the capacities that allow them to interact successfully with their biological, physical, and social environments.”^7^ (pages 32 and 33)

Several features of this definition are noteworthy. First is the continued conceptualization of health as a positive construct – more than the absence of illness or disease. Second, it incorporates the special characteristics, particularly rapid development and continuous change throughout childhood, as essential components of health. It also considers all the many influences that interact over time in different ways as children develop and change, and it acknowledges the ways children interact with their specific environments and the long-term implications of these environmental factors. This definition underscores the long reach of child health into adulthood underscoring that the health of children has profound effects on the health of the adults they will become. It acknowledges that the manifestations of health may vary for different communities and cultures and encompasses all aspects of health: physical, emotional, cognitive, and social health.

## Domains of health

So how did the committee conceptualize the measurement of child health? First, it should be acknowledged that most commonly used measures are actually proxies of health or measures of only one aspect of the more complex construct embodied in the definition. For example, we might use body mass index to define obesity, or measure only cognitive functioning on a psychological test.

The model also emphasized the importance of tracking data on children’s health or aspects of their health on trajectories in a manner that is like the ways weight, length, are tracked. One cannot know the meaning of most isolated measures without knowing their place on a trajectory. For example, it is impossible to know if 20 pounds is a healthy weight or not without knowing the child’s age and prior weights. Similarly, one would have trouble determining the developmental health of child who speaks in 3-word sentences without knowing his age and whether his language was previously more or less advanced. Assessing trajectories was viewed as an essential part of efforts to improve children’s health. This requires longitudinal data.

Nevertheless, the committee conceptualized three domains of health that should be the basis for measuring child health: *Health conditions*: disorders or illnesses of body systems; *Functioning*: manifestation of health on an individual’s daily life, and *Health potential*: development of assets and positive aspects of health, such as resilience, competence, capacity, and developmental potential.

In considering the measurement of health conditions, it is important to note that these conditions can either be acute or chronic. Health conditions are the most traditional way of measuring health –or its absence. These conditions are usually inventoried by clinician diagnoses or by questionnaires inquiring about specific conditions or diseases. Those that are chronic can be assessed using two major approaches. The first is using a list or inventory of individual conditions. However, the list of such conditions is extensive because of the large number of uncommon disorders, and no list can be complete and be feasible to administer. Unfortunately, evidence shows that the more examples that are provided on a list, the more likely people will respond to the option of “or any other condition.” This finding is clearly counterintuitive and limits the utility of a list approach.

Another method of inventorying chronic conditions depends on a non-categorical or generic approach.^9–11^ This approach explores the consequences of conditions, as well as their duration, based on a noncategorical definition.^12^ The definition includes having a condition that lasts or is expected to last a year and having at least one of three types of consequences of conditions: Increased use of health care beyond the usual for age; dependence on a compensatory mechanism or assistance to function in a typical way; or the presence of functional limitations.^12^ Three instruments that operationalize that definition have been developed and are in use.^13–16^

This approach allows the identification of children with ongoing conditions without having to name the condition. A non-categorical approach is now incorporated into several national surveys using the shortest of these instruments, the CSHCN Screener.^15^ It is used to track both the number of children with conditions and disparities in the ways in which care is delivered to children both with and without ongoing conditions.

Functioning has been defined by the International Classification of Functioning, Disability and Health as “an umbrella term for body function, body structures, activities and participation. It denotes the positive or neutral aspects of the interaction between a person’s health condition(s) and that individual’s contextual factors (environmental and personal factors)” (https://www.cdc.gov/nchs/data/icd/icfoverview_finalforwho10sept.pdf). Functioning is viewed as the way in which an individual can do things and is the final expression of health of individuals. One strength of this type of measure is that it can assess the consequences of many coexisting conditions and both conditions and their treatment. This is not something that is possible to assess when considering conditions as proposed above. Even when multiple conditions are inventoried individually, it does not give any indication of their combined effect on the child’s health. Also, in some instances, there are more symptoms or impairments from the treatment than from the condition itself, such as when encountering serious side effects from chemotherapy, during treatment or when the original condition itself is in remission. There are few other ways in which to get the type of summative information that can be obtained by assessing functioning.^17^

There are relatively few measures of functional limitations specifically developed for children. Many of the measures of functioning in the past have focused on gross motor functioning and some of them measure only one type of functioning, such as cognition, which is measured by a range of psychological tests. More recently there have been attempts to develop more comprehensive measures. Few of them work across populations, culture, levels of health/disability and ages. Some more comprehensive measures are age specific, such as measures of development or of school readiness.^18^ In general, such measures assess a range of skills including independence, physical, social, cognitive, emotional, and language skills. Nevertheless, there are few functional measures that work across populations, cultures, levels of health/disability and ages. Among the range of measures that are broader and in use are FSII (R)^19^; Wee FIM^20^ and Functional Status Scale^21^ and health quality of life measures.^22^ Each of them has a different focus and measures different aspects of functioning.

There are even fewer measures of health potential, but this is an important area for future research. This domain is critical to improving understanding of why some children experiencing a major stress are able to bounce back and overcome the trauma, while others are stunted in their further development, or never rebound at all. Some areas that are included are resilience, problem solving ability, resistance to illness, immunization status, ability to develop positive peer relations, and physical fitness. How these factors fit together and become protective is an area worthy of exploration, but clearly some children differentiate themselves from others by their ability to rebound from adversity or illness, while others suffer long-term consequences of poor health and well-being.

Finally, it is important to acknowledge that the entire field of measurement is complicated by the fact that many children, especially younger ones cannot reliably respond to questionnaires on their own. As a result, most measures require responses from other individuals, typically caregivers. Others are completed by clinicians. Their biases and differences in their frames of reference may further complicate all these measures.

## Factors influencing health

In exploring the issue of risk and protective factors that influence and affect health status, the committee realized that many known factors did not fall neatly into either category. In some cases, it was because they may be both risks and protective depending on the context (i.e: peer groups). In others, it’s effect was dependent on the level of exposure, as might be characterized by iron on iodine, both of which can cause problems if they are insufficient or be toxic if exposure is excessive.

Rather than think of them in terms of risk or protective factors, the committee chose to conceptualize the factors that affect child health as *influences*, since many may be both risks and protective, depending on the context and level of exposure.

The influences were grouped into six categories following the model of Healthy People 2010, which was the operant model at the time.^23^ One objection to that model was that it was very linear, something that seemed at odds to the ways in which influences are understood to interact. However, the committee thought that the major categories or domains in the model that affected health were sound. These components were: biology, behavior, physical environment, social influences, services, and policy. Another significant modification of the Healthy People 2010 model was the considerable expansion of services and policy domains beyond those of health policy and health services, which was the original intent of the 2010 model. This is because the committee recognized that a wide range of services (e.g. education, welfare, and sanitation services) and policies (e.g. tax, law enforcement, road safety and environmental policies) have considerable impact on child health. Each of these categories was conceptualize as having many elements within them. A partial list of components of these domains is shown in Table 1. Both within the groupings and across groupings these influences interact with one another and their relative importance changes over time and through development. Some of these changes are predictable and others depend on what the individual child experiences. For example, in early childhood the family is probably the most important social influence, while later in development other components, such as the community and the peer group, have greater impact. In terms of unique experiences, changes in the family composition or family dislocation, illness or toxic exposures may have great impact in one child’s development, in contrast with those who experience long term stability.

**Table 1:** A partial list of influences affecting child health.

Biology
Genes
Gene expression
Body stores
Body composition
Prenatal influences
Infections, illness, trauma
Behavior
Emotions
Attitudes
Beliefs
Cognition
Physical environment
Air, water, and food quality
Toxins
Housing
Radiation
Noise
Schools
Traffic
Built environment
Weather
Social environment
Family: composition, size
Socioeconomic status
Mental and physical health
Parenting style
Relations with caregivers
Peers
Community
Culture
Race, ethnicity, discrimination, segregation
Educational opportunity
Media
Services
Health
Education
Social welfare, safety net
Sanitation
Policies
Health care, insurance
Education
Social welfare
Taxation
Environmental

## A new model

Altogether various influences interact over time and throughout development in a way that can be compared to and visualized as being like a kaleidoscope. That is to say, the patterns that emerge are partly determined by the initial constellation of factors at the time of the child’s birth. All prior exposures are embedded in his or her biology at birth and are incorporated into the initial template. But two individuals with different initial patterns will react differently to subsequent influences, even when they are exposed to the identical ones. Moreover, influences that are experienced by the individual at different stages of development will also have discrete effects, depending on when they are experienced. As a result, two children with different preexisting templates may react differently and their subsequent health will reflect those differences.

**Fig. 1:**
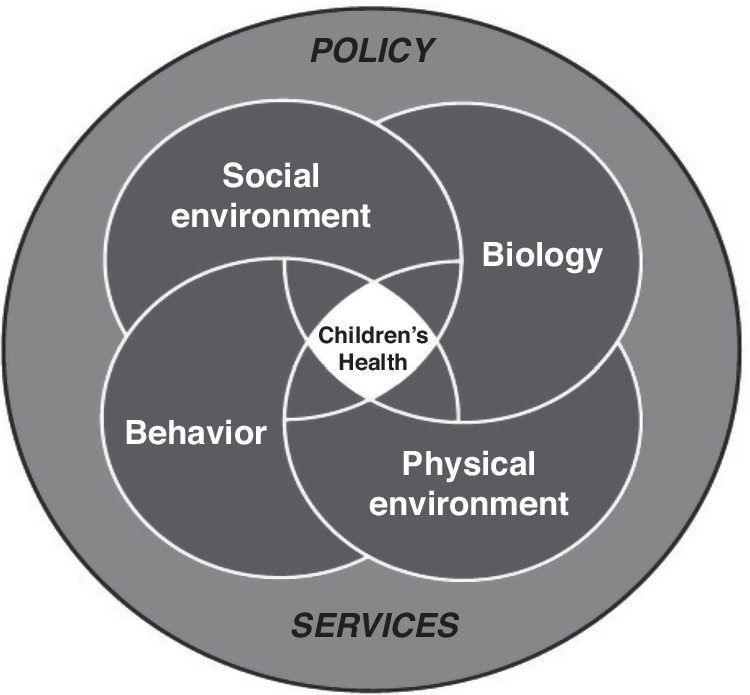
Multiple interacting influences.

A picture of the component influences at any given time can be visualized as a Venn diagram (Fig. 1). Within each component, there are many subcomponents, as discussed in the section on influences, and each of those subcomponents may be of different importance at a given time and stage. They may be viewed as mini kaleidoscopes within the domain and are also similar to the whole domain in that they vary in their importance throughout development.

**Fig. 2:**
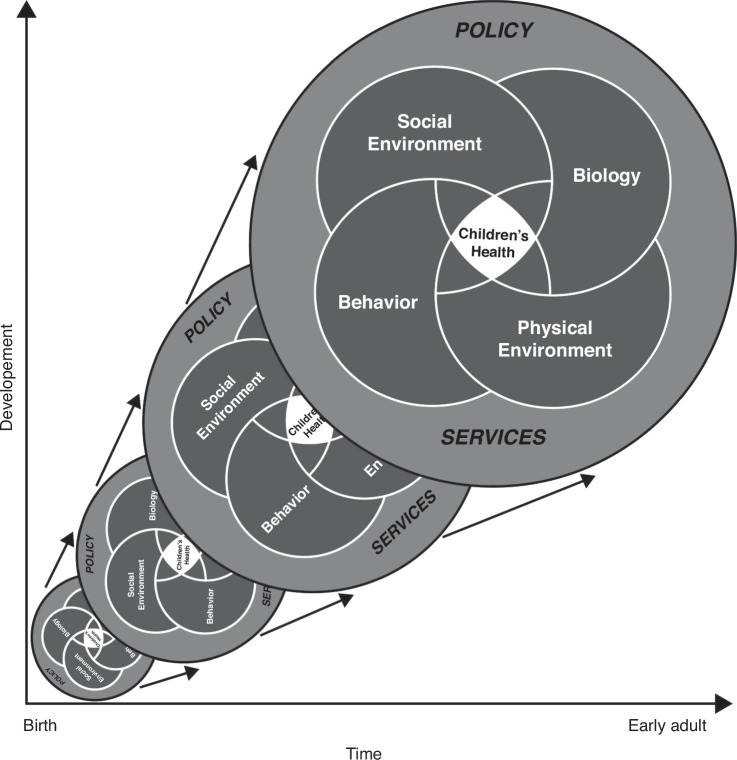
Model of children’s health and its influences.

As things change during a child’s development and over time, the kaleidoscope changes, depending on how the influences affect the individual or group of children (Fig. 2). The ways in which influences of various types affect a given child will depend on the arrangements of the preexisting template at the time of the new experience. This is like giving a twist to the crystals in the kaleidoscope, in which different sets of crystals will produce differing patterns.

Additionally, the committee also recognized that there are some periods of time that are critical or sensitive and have magnified impact on health and development. Critical periods are ones in which an influence has a determinative effect on health, such as early prenatal exposure to Thalidamide during a critical period of embryogenesis, while sensitive periods are ones in which there is increased vulnerability, but no absolute effect. During these periods, exposure to certain influences has a more significant impact. For example, children who are not exposed to language in early infancy, may not recoup that loss completely, while that same lack of exposure later in development may have a far smaller effect. Similarly, parental separation or death may have different consequences depending on both the child’s template at the time of the occurrence and the age at which the trauma is experienced.

## Nature vs nurture

For many generations people have argued about the role of nature versus nurture in determining health outcomes. However, in the last several decades this debate finally has some important answers, and we are finally beginning to understand how environments “get under our skin.” Since the mapping of the human genome, we have learned that the environment affects and alters the expression of our genes mainly by upregulating and down regulating them through epigenetic mechanisms. At other times exposures to specific influences actually interfere with gene replication and expression in a more deterministic fashion, as when there is exposure to radiation that alters the genes themselves.

Moreover, we now understand that adverse childhood experiences produce toxic stress and that when the allostatic load becomes too great, it produces changes in gene expression through epigenetic mechanisms. The ensuing changes affect multiple body systems including the brain, autonomic, neuroendocrine, immune, cardiovascular, and gastro-intestinal systems.^24,25^ These changes have been associated with chronic inflammation, something that may affect long-term health and survival and sometimes can even be passed to the next generation, as has been shown for the effects of racism. Epidemiologic studies have long supported that increasing numbers of childhood exposures to these forms of stress are associated with physical and mental illness and premature death in adulthood.^26,27^

It is important to also acknowledge the critical role that caregivers play in nurturing children and in buffering them against the noxious effects of stresses. This nurturance and buffering effect is something that is critical in helping children to thrive in spite of influences that threaten their health. It is also likely that those buffers are of special importance at times of transition in growth and development.

## Implications

The implications of the committee’s definition and model of health are far reaching. One inherent implication of the model is that we can never measure all the factors that influence child health in any single study. As a result, it brings a new perspective to some of our efforts to interpret research data. For example, if two studies of outcomes of very low birth weight infants come to somewhat different conclusions about predictors of outcome, our tendency has been to try to determine which study was flawed and which was more reliable. Perhaps, instead we should question whether the subjects differed in some unmeasured, but significant, way that influenced their outcome.

The model also emphasizes that health does not derive primarily from medical care. In doing so it brings into question the ways in which our society divides budgets for the many kinds of services and policies that contribute to health and healthy development. The effects of decisions in these domains often omit consideration of their impact on children’s health and well-being. Some have suggested that we should have a process like our consideration of environmental impact for projects that would consider child health impact when new projects or policies are put into effect.

Finally, the model underscores the long reach of childhood influences on adult health. This is far more appreciated now, than at the time of the committee report, because of several factors. First is the increased understanding of epigenetics and the long-term implications of changes in gene expression. Another factor is the growing literature on the effects of adverse childhood experiences (ACES). It is now unquestionable that these societal issues impact both child wellbeing and adult health and survival. Our awareness of these factors has also led to far more consideration of other social factors, and to the appreciation of the influence of social determinants of health. These include economic stability, education access and quality, health care access and quality, neighborhood and built environment, social and community context (https://health.gov/healthypeople/priority-areas/social-determinants-health) – a list quite like those in the Committee’s model.

The growing awareness of the long reach of child health is important for the field of pediatrics, which has long suffered from a lack of investment. This is a result of the degree to which finances have driven investment in health and health care. In general child health costs are so much lower than the costs of adult care, except for care of the very low birth weight infant and certain malignancies. As appreciation grows for the importance of environmental factors during childhood on ultimate health, we can hope that investments increase in relatively low-cost preventive measures that may alter longer term outcomes.

## Future projections

Given our increased knowledge about the impact of environment and life events on children, we would be remiss if we did not highlight the changing nature of the world in which we live. The numerous wars around the world are massively disrupting children’s lives and causing mass migration. The COVID pandemic caused millions of deaths, including those of many caregivers, and world-wide disruption of daily life with loss of educational and social opportunities for countless numbers of children. Many of these losses appear to be having long-reaching impact on their education, development, and mental health.^28^ Additionally, the direct and indirect effects of climate change, which is making some areas of the globe less habitable, and subjecting others to catastrophic weather event, fires, and floods, are producing massive dislocations. Many of these influences are affecting the children who are already most vulnerable.

When children experience these catastrophes, it has lasting effects on their health and developmental trajectories. In addition to the frequent events themselves, they are often accompanied by loss of caregivers, whose protection is so important to helping children deal with stresses, and to loss of routines, which provide stability and a sense of normalcy. Additionally, they often lose educational opportunities that would enable them to develop skills that would improve their future welfare. Even when there are no direct physical or observable injuries, all these factors increase children’s allostatic load and are embedded in their gene expression, causing inflammation and premature aging of many body systems, and setting them up for future poor health. It is important for the child health community to do all that it can to help buffer these effects and to help inform policy makers of their long impacts and costs to the individual and to society. The definition and model inform the child health community that our failure to do so is likely to be accompanied by a generation whose health and well-being is in peril.

## Conclusions

We believe that the definition that the committee adopted and the model of how health evolves has had a major impact on thinking in the field. To some extent it forecast the CDC’s Health People 2020’s model of health “that recognized a life stages perspective. This approach recognizes that specific risk factors and determinants of health vary across the life span. Health and disease result from the accumulation (over time) of the effects of risk factors and determinants” (https://wayback.archive-it.org/5774/20220413162937/https://www.healthypeople.gov/2020/leading-health-indicators/Leading-Health-Indicators-Development-and-Framework). The emphasis on child development may have had some role in helping other clinicians to focus on the fact that development does not stop when one reaches adulthood. It is entirely compatible with the Healthy People 2030’s goal to “Create social, physical, and economic environments that promote attaining the full potential for health and well-being for all”^29^ and can serve as a guiding principle for pediatrics and our society. It suggests that society should want to invest in children because they are our nation’s most important resource. The definition of child health presented by the committee has many useful principles that can guide our research, clinical care, and policies to try to protect long-term thriving of the maximum number of children. It is one that can continue to guide our work for many decades to come.
